# The effect of a novel curcumin derivative on pancreatic islet regeneration in experimental type-1 diabetes in rats (long term study)

**DOI:** 10.1186/1758-5996-5-75

**Published:** 2013-11-26

**Authors:** Mohamed T Abdel Aziz, Mohamed F El-Asmar, Ameen M Rezq, Soheir M Mahfouz, Mohamed A Wassef, Hanan H Fouad, Hanan H Ahmed, Fatma M Taha

**Affiliations:** 1 Unit of Biochemistry and Molecular Biology, the Medical Biochemistry Department, Faculty of Medicine, Cairo University, Kasr El Aini, Cairo, Egypt; 2 Medical Biochemistry Department, Faculty of Medicine, Ain Shams University, Cairo, Egypt; 3 Pathology Department Faculty of Medicine, Cairo University, Cairo, Egypt

**Keywords:** Curcumin derivative, Type I diabetes, Pancreatic islet regeneration, Insulin secretion

## Abstract

**Background:**

Several studies highlight curcumin’s benefit as a hypoglycemic agent, however; a limited number of reports present the importance of curcumin in improvement of pancreatic islets in diabetes. The aim of the present study is to evaluate the antidiabetic effect of a novel curcumin derivative and its effect on pancreatic islet regeneration in type I diabetes-induced by STZ.

**Materials and methods:**

Rats were divided into diabetic rats and diabetic rats treated orally with the novel curcumin derivative (NCD) for 40 days. Fasting blood samples were withdrawn periodically from all rats to estimate plasma glucose, insulin and C-peptide for 10 months. Histopathology was performed to allow the assessment of pancreatic islet morphology. Insulin and CD105 were detected immunohistochemically.

**Results:**

In diabetic rats, the plasma glucose, insulin and C-peptide levels remained within the diabetic range for about 4 months, after which a gradual decrease in glucose and increase in insulin and C-peptide was observed, which reached almost normal levels after 10 months. NCD treated diabetic rats showed significantly lowered plasma glucose and increased plasma insulin and C-peptide levels. This was followed by a further significant decrease in plasma glucose and increase in plasma insulin and C-peptide after two months from oral administration of the NCD. The plasma insulin and C-peptide continued to increase for ten months reaching levels significantly higher than the basal level. Histopathological examination of diabetic rat pancreas revealed absence of islets of Langerhans, minimal adipose tissue infiltration and localized lymphocytic infiltrates. However, after 6 months of induction of diabetes, rat pancreas showed the appearance of small well formed islets and positive insulin cells but no CD105 positive cells. NCD treated rats showed the appearance of primitive cell collections, large insulin positive cells and CD105 positive cells in the adipose tissue infiltrating the pancreatic tissues. This was followed by the gradual appearance of insulin positive cells in the islets while, CD 105 positive cells remained in the adipose tissue. After 5 and 10 months from the onset of diabetes, rat pancreas showed, well developed larger sized islets with disappearance of primitive cell collections and CD 105 positive cells. Also, insulin positive islets of variable size with disappearance of insulin positive cells in adipose tissue were detected.

**Conclusion:**

The NCD possesses antidiabetic actions and enhanced pancreatic islets regeneration.

## Background

The prevalence of diabetes for all age-groups worldwide was estimated to be 2.8% in 2000 and will rise to 4.4% in 2030. The total number of people with diabetes is projected to rise from 171 million in 2000 to 366 million in 2030 [1].

Diabetes mellitus type I is an autoimmune disorder caused by lymphocytic infiltration and β-cells destruction within the pancreatic islets of Langerhans. The pancreatic β-cells are lost in number and volume, leading to severe permanent insulin deficiency [2].

Transplantation therapies for type 1 DM include whole organ transplantation [3], transplantation of isolated islets [4,5] and regeneration therapy [6]. Although the transplantation of both a whole organ and isolated islets has been successfully used in the clinical treatment of type 1 DM, a shortage of donors limits the widespread use of this treatment modality. Additionally, the quality of a donor’s pancreas is an important criterion for islet isolation [7]. Therefore, regeneration of pancreatic islets is certainly a worthwhile therapeutic goal that would substantially ameliorate diabetes and lessen its complications [8].

An alternative strategy to treat diabetes is the use of various plant extracts and herbal biomolecules, due to their hypoglycemic effects. Detailed investigation of these biomolecules has revealed that some of them cause a regeneration of β-cells, thus causing a reversal of diabetes in human and non-human subjects [9-11].

Curcumin, the naturally occurring yellow pigment isolated from the rhizomes of the plant Curcuma longa, has been shown to possess antioxidant, anti-tumor, and anti inflammatory properties [12,13]. At the cellular and molecular levels, such effects for curcumin are mediated by free-radical scavenging, up-regulation of defense proteins; such as heme oxygenase-1 (HO) and reduced glutathione, and suppression of pro-inflammatory/pro-apoptotic cytokines/transcription factors; like TNF-α and NF-κB [14,15].

The potential of curcumin as a hypoglycemic agent has been studied in animals [16,17]; yet with conflicting results.

Abdel Aziz et al. [18] reported that insulin secretion, HO-1 gene expression and HO activity were significantly increased when rat isolated islets of Langerhans were incubated in curcumin.

Abdel Aziz et al. [19] studied the antidiabetic effect of a water soluble curcumin derivative (NCD) containing only 3.0% curcumin and its effects on diabetes-induced reactive oxygen species (ROS) generation and lipid peroxidation in experimental type- 1 diabetes mellitus. They proved that the NCD had the ability to decrease plasma glucose and increase plasma insulin levels significantly in diabetic rats. NCD also improved the lipid profile and oxidative status, proved by decreasing lipid peroxides (malondialdehyde) in pancreas, liver & aorta.

Because of the poor bioavailability of pure curcumin, a new water soluble curcumin derivative was used in this study.

The aim of the present study is to evaluate the antidiabetic effect of a novel curcumin derivative (NCD) and its effect on pancreatic islet regeneration in type I diabetes.

## Materials and methods

This study was performed at the Unit of Biochemistry and Molecular Biology at The Medical Biochemistry Department, Faculty of Medicine, Cairo University, Egypt. The curcumin derivative was presented free of charge to the participating researchers as a personal nonprofit scientific donation to help advancement of cooperation in national medical research. The novel derivative, with 53.21% curcumin content is registered as international patent protected by the rights of “The Patent Cooperation Treaty” and are the personal property of their inventors (PCT/EG2008/000044, WO 2010/057503, Regional phase European Patent Application No. 08878223) [20]. The novel water-soluble curcumin derivative with conserved natural functional groups is [1,7-bis (5-carboxyphenylazo-4-hydroxy-3-methoxyphenyl)1,6-heptadiene-3,5-dione]. This novel water-soluble curcumin derivative with conserved natural functional groups was developed in our laboratories through covalent modification of the curcumin molecule on sites remote from its natural functional groups.

### Experimental animals

The experiments were performed on forty adult rats weighing 150 to 200 gm obtained from an inbred colony (Curl: HEL1) at the Kasr Al-Aini animal experimental unit, Faculty of Medicine, Cairo University. These animals were kept in an environment with controlled temperature (25°C), humidity (45-75%) and 12:12 h light:dark cycle. All animals were fed ad libitum and had free access to water. All the ethical protocols for animal treatment were followed and supervised by the animal facilities, Faculty of Medicine, Cairo University. All animal experiments received approval from the Institutional Animal Ethics Committee.

### Induction of diabetes mellitus

The animals were acclimatized for 1 week before initiation of the experiment. Diabetes mellitus was induced in forty rats by a single intraperitoneal injection of streptozotocin (STZ) dissolved in 0.1M sodium citrate buffer, pH 4.5, at a dose of 50 mg/kg. After 72 h, fasting blood glucose levels were monitored and animals with blood glucose levels >200 mg/dL were considered diabetic.

### The forty rats were divided into two groups

Group I: Twenty diabetic rats as a control group receiving no medications.

Group II: Twenty diabetic rats treated with the novel curcumin derivative (NCD):

They received the NCD orally and in a daily dose of 150 mg/Kg body weight for forty days only starting from the day after the onset of diabetes. (This dose is equivalent to 80 mg/Kg body weight of pure curcumin).

Fasting blood samples were withdrawn periodically from both groups from the tail vein to estimate plasma glucose, insulin and C-peptide.

### Plasma glucose estimation

Blood was collected from the tail vein into tubes containing fluoride. Plasma samples were separated by centrifugation at 3000 rpm for 10 min. Plasma glucose was measured by the glucose oxidase method [21] using a commercially available kit, Cat No 3728 (Diamond, Egypt).

### Plasma insulin estimation

Plasma insulin was assayed by a commercially available Enzyme-linked immunosorbent assay (ELISA) kit, Cat No EIA - 2048 supplied by DRG Diagnostics (GmbH, Germany) [22].

### Assessment of C-peptide

C-peptide was assessed by a commercially available Enzyme-linked immunosorbent assay (ELISA) kit,Cat No 2725-300A supplied by Monobind Inc(Lake Forest, Ca, USA) [23].

### Histological and immunohistochemical analysis

Pancreata were excised, and then fixed in 10% neutral buffered formalin. Tissues were then processed for paraffin embedding, subsequent serial sectioning, and stained with hematoxylin/eosin (H&E) to allow the assessment of pancreatic islet morphology in the studied groups.

Insulin was detected immunohistochemically by monoclonal antibodies, Insulin Ab-6 (INSO4 + INSO5) monoclonal antibody, Cat No MS-1379-P : (200 μg/mL); Purified antibody with BSA and azide); supplied by Thermo Scientific (Fremont, USA).

CD 105 was detected immunohistochemically by monoclonal antibodies, CD 105/Endolgin monoclonal antibody, Cat No MS –1290-P: (200 μg/mL); Purified antibody with BSA and azide); supplied by Thermo Scientific (Fremont, USA).

### Statistical analysis

Unpaired Student's *t*-test was used for testing statistical significance of difference between every 2 groups using the PC software “Statistica version 8.0” of Statsoft Inc., USA. Paired Student’s t*-* test was used for testing statistical significance of difference between time intervals in the same group. Data were presented as mean ± SD. The differences between groups were considered to be significant at *p* <0.05.

## Results

After induction of diabetes, 32 rats were remaining. These rats were subgrouped into 16 diabetic and 16 diabetic rats receiving the (NCD). Table 1 shows the studied parameters in the groups at basal level and after induction of diabetes by STZ.

**Table 1:** Studied parameters at basal levels & after STZ-induced diabetes

**Parameter**	**Basal (N=40)**	**Diabetic (N=32)**	**p value**
Glucose (mg/dL)	92.83 ± 5.39	285.01 ± 54.64	<0.001
Insulin (μg/L)	4.68± 0.84	0.65 ±0.14	<0.001
C-peptide (ng/L)	2.89 ± 0.6	0.45 ± 0.11	<0.001

Data were presented as mean ± SD.

p value (significant difference by unpaired student-*t* test).

### Plasma glucose levels

**Figure 1:**
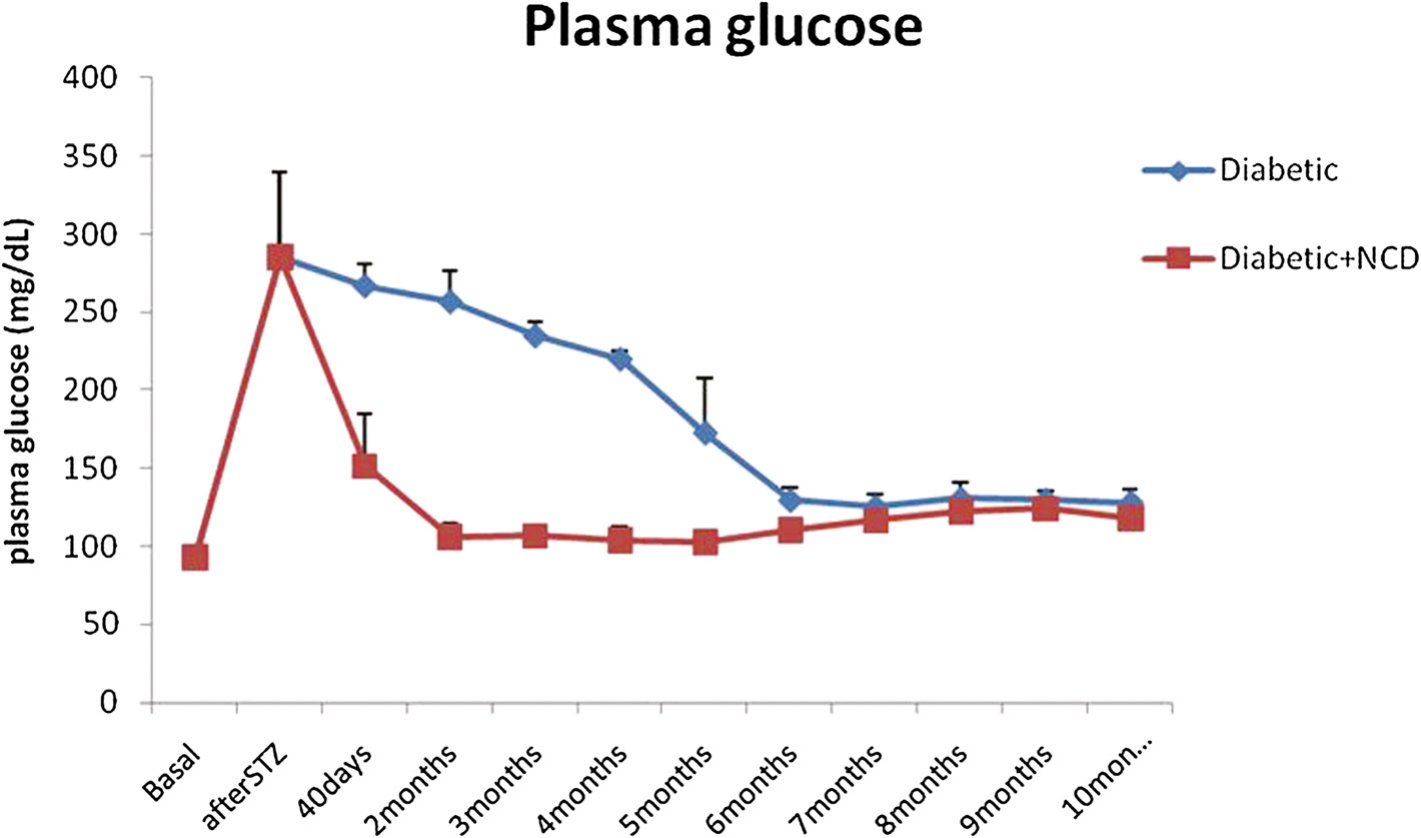
Changes in mean fasting plasma glucose levels in STZ-induced diabetic rats and diabetic rats receiving NCD.

Figure 1 shows the changes in the fasting plasma glucose levels in Group I and Group II rats.

The mean plasma glucose level of diabetic rats was significantly increased (p < 0.001) compared to the basal level. The rats showed hyperglycemia (plasma glucose > 200 mg/dL) 72 hrs after STZ treatment. The mean plasma glucose level remained significantly elevated after forty days after the onset of diabetes. This was followed by a gradual decrease in the mean plasma glucose levels which continued till 10 months after the onset of diabetes but was still significantly higher (p < 0.001) than the basal level (128.2 ± 8.9 mg/dL). The mean plasma glucose level of diabetic rats receiving the NCD was significantly increased (p < 0.001) compared to the basal level. The rats began to show hyperglycemia (plasma glucose > 200 mg/dL) 72 hrs after STZ treatment. The NCD was administered for 40 days after the onset of diabetes. After these 40 days, the mean plasma glucose of diabetic rats (151.51 ± 32.73 mg/dL) showed a significant (p < 0.001) decrease compared to the diabetic level (285.01 ± 54.64 mg/dL) which was followed by a further significant (p < 0.001) decrease (106.09 ± 8.61 mg/dL) after two months from the onset of diabetes. This decrease in plasma glucose level was maintained for ten months but was still significantly higher (p < 0.001) than the basal level (117.95 ± 9.05 mg/dL).

### Plasma insulin levels

**Figure 2:**
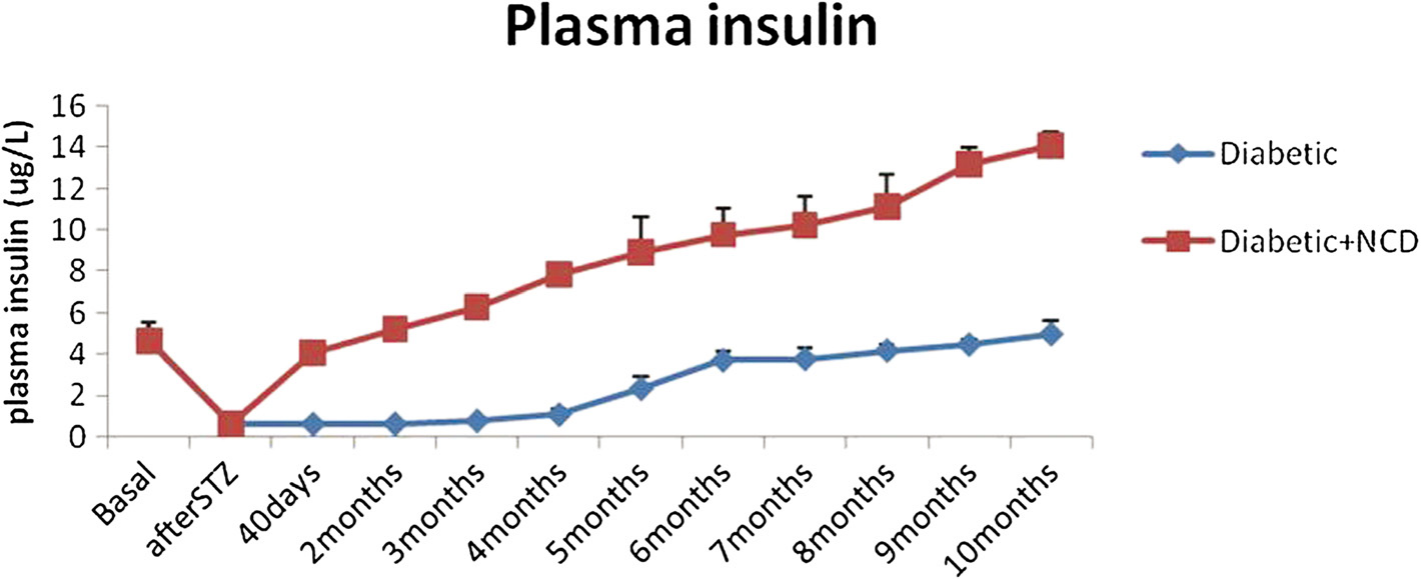
Changes in mean fasting plasma insulin levels in STZ-induced diabetic rats and diabetic rats receiving NCD.

To gain insights into the insulin secretory capacity of pancreatic islets and its relationship with plasma glucose, we monitored fasting plasma insulin levels in diabetic rats and diabetic rats receiving the NCD. Figure 2 shows the changes in the fasting plasma insulin levels in Group I and Group II rats. The mean plasma insulin level of diabetic rats was significantly decreased (p < 0.001) compared to the basal level. This was followed by a gradual increase in the mean plasma insulin levels which increased significantly after 4 months (1.12 ± 0.26 μg/L) and reached almost normal levels 10 months after the onset of diabetes (5 ± 0.6 μg/L). The mean plasma insulin level of diabetic rats receiving the NCD was significantly decreased (p < 0.001) compared to the basal level. After the 40 days of administration of NCD, the mean plasma insulin level of diabetic rats (4.09 ± 0.45 μg/L) showed a significant (p < 0.001) increase compared to the diabetic level (0.65 ± 0.14 μg/L) which was followed by a further significant (p < 0.001) increase (5.26 ± 0.55 μg/L) after two months from the onset of diabetes. The plasma insulin levels continued to increase for ten months (14.11 ± 0.66 μg/L) reaching levels significantly higher than the basal level (p < 0.001).

### Plasma C-peptide levels

**Figure 3:**
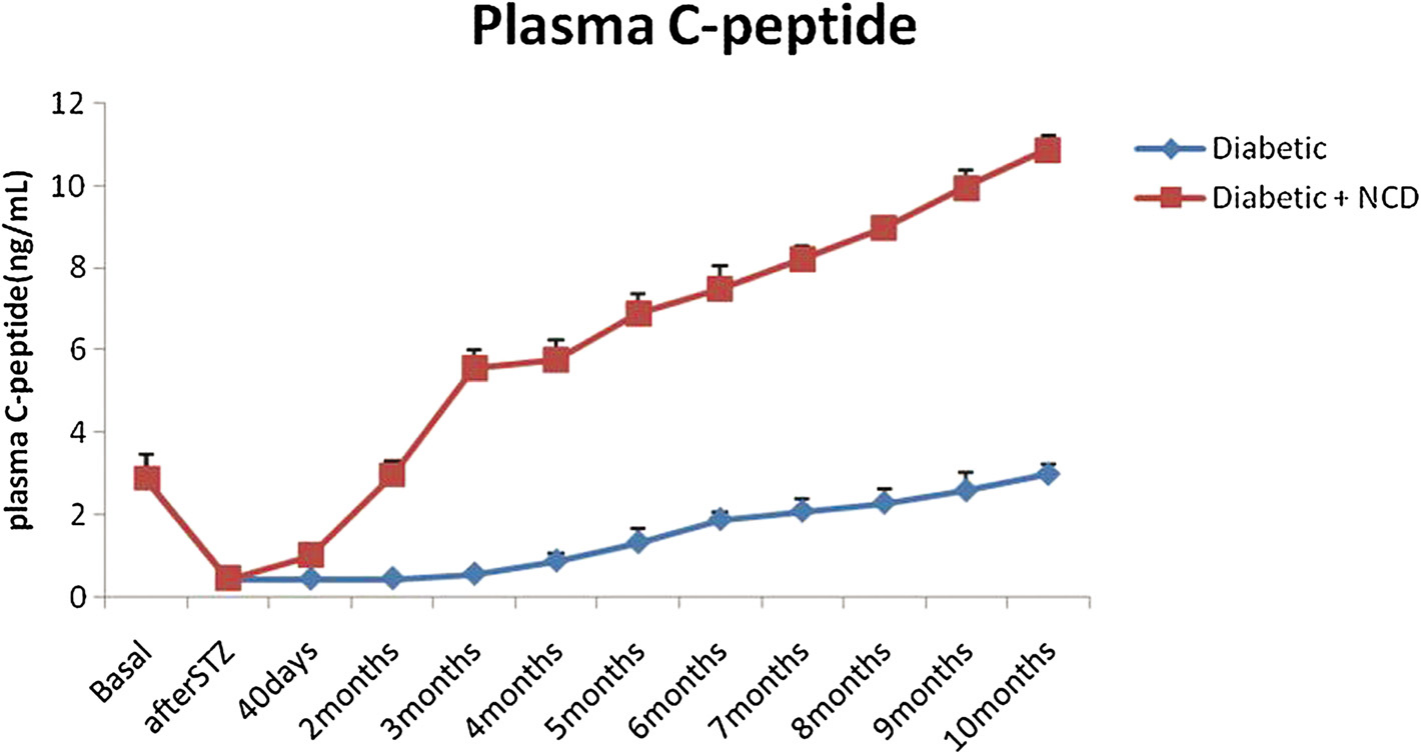
Changes in mean fasting plasma C-peptide levels in STZ-induced diabetic rats and diabetic rats receiving NCD.

Figure 3 shows the changes in the fasting plasma C-peptide levels in Group I and Group II rats: The mean plasma C-peptide level of diabetic rats was significantly decreased (p < 0.001) compared to the basal level. The mean plasma C-peptide levels correspond to the fasting plasma insulin levels in both diabetic rats and diabetic rats receiving NCD.

### Histopathological and immunochemical results

**Figure 4:**
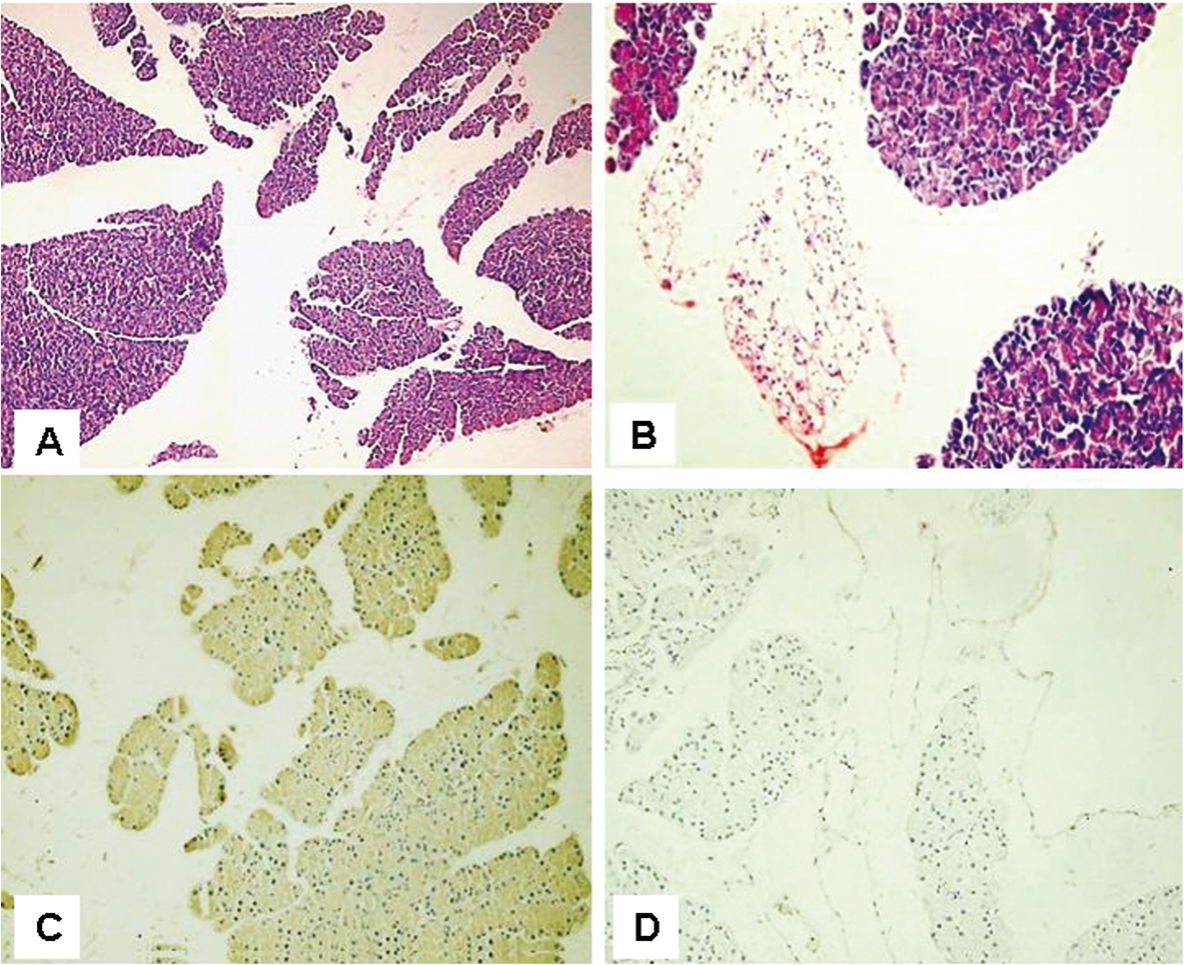
**Micrographs of diabetic rat pancreas after 2 months of induction of diabetes. (A)** Rat pancreas stained with Hematoxylin and Eosin (H&E) showed mostly exocrine pancreatic tissue with no islets of Langerhans and minimal adipose tissue infiltration(X100) and **(B)** Localized lymphocytic collections (X200) **(C)** Diabetic rat pancreas stained with insulin antibody with no positive insulin secreting cells (X200) **(D)** Diabetic rat pancreas stained with CD 105 with no evidence of CD 105 positive cells (X200).

Histological examination of the H&E-stained paraffin sections of rat pancreas subjected to streptozotocin (after 2 months from induction of diabetes) showed mostly exocrine pancreatic tissue with no islets of Langerhans and minimal adipose tissue infiltration (Figure 4A) and localized lymphocytic collections(Figure 4B). Diabetic rat pancreas stained with insulin antibody showed absence of islets of Langerhans and no positivity with antiinsulin in either pancreatic tissue or fat (Figure 4C). Diabetic rat pancreas stained with CD 105 antibody showed no evidence of CD 105 positive cells in either pancreatic tissue or fat (Figure 4D).

**Figure 5:**
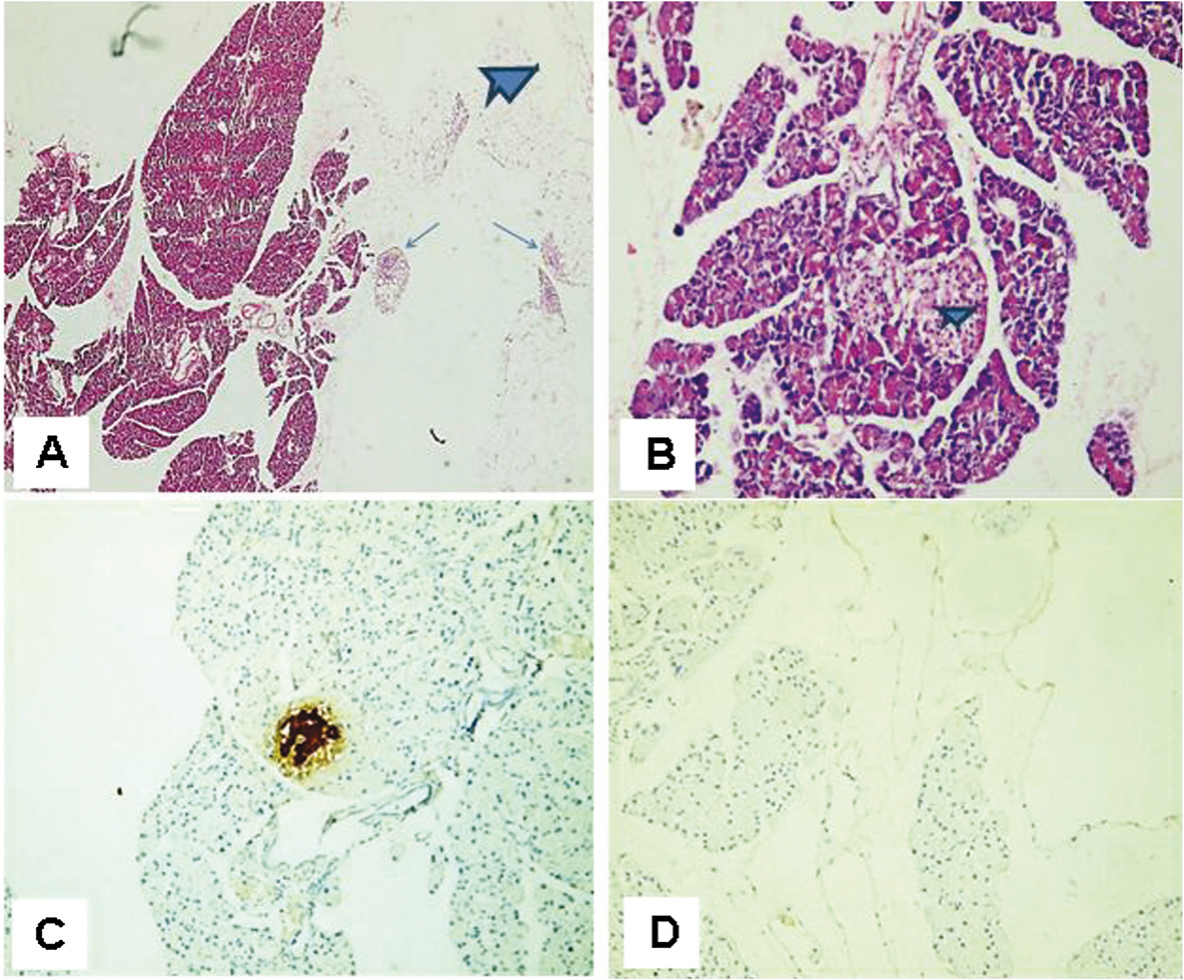
**Micrographs of diabetic rat pancreas after 6 months of induction of diabetes. (A)** Rat pancreas stained with H&E showed increased adipose tissue infiltration (star) and localized lymphocytic collections (arrows) (X40) **(B)** Rat pancreas with small well-formed islet (star) **(C)** Diabetic rat pancreas stained with insulin antibody showed small well-formed islet (brown) secreting insulin. (X200) **(D)** Diabetic rat pancreas stained with CD 105 antibody showed no evidence of CD 105 positive cells in either pancreatic tissue or fat(X200).

After 6 months from the induction of diabetes, rat pancreas showed increased adipose tissue infiltration and localized lymphocytic collections (Figure 5A) and appearance of a small well-formed islet (Figure 5B). While the diabetic rat pancreas stained with insulin antibody showed a small well-formed islet secreting insulin (Figure 5C), diabetic rat pancreas stained with CD 105 antibody showed no evidence of CD 105 positive cells in either pancreatic tissue or fat (Figure 5D).

**Figure 6:**
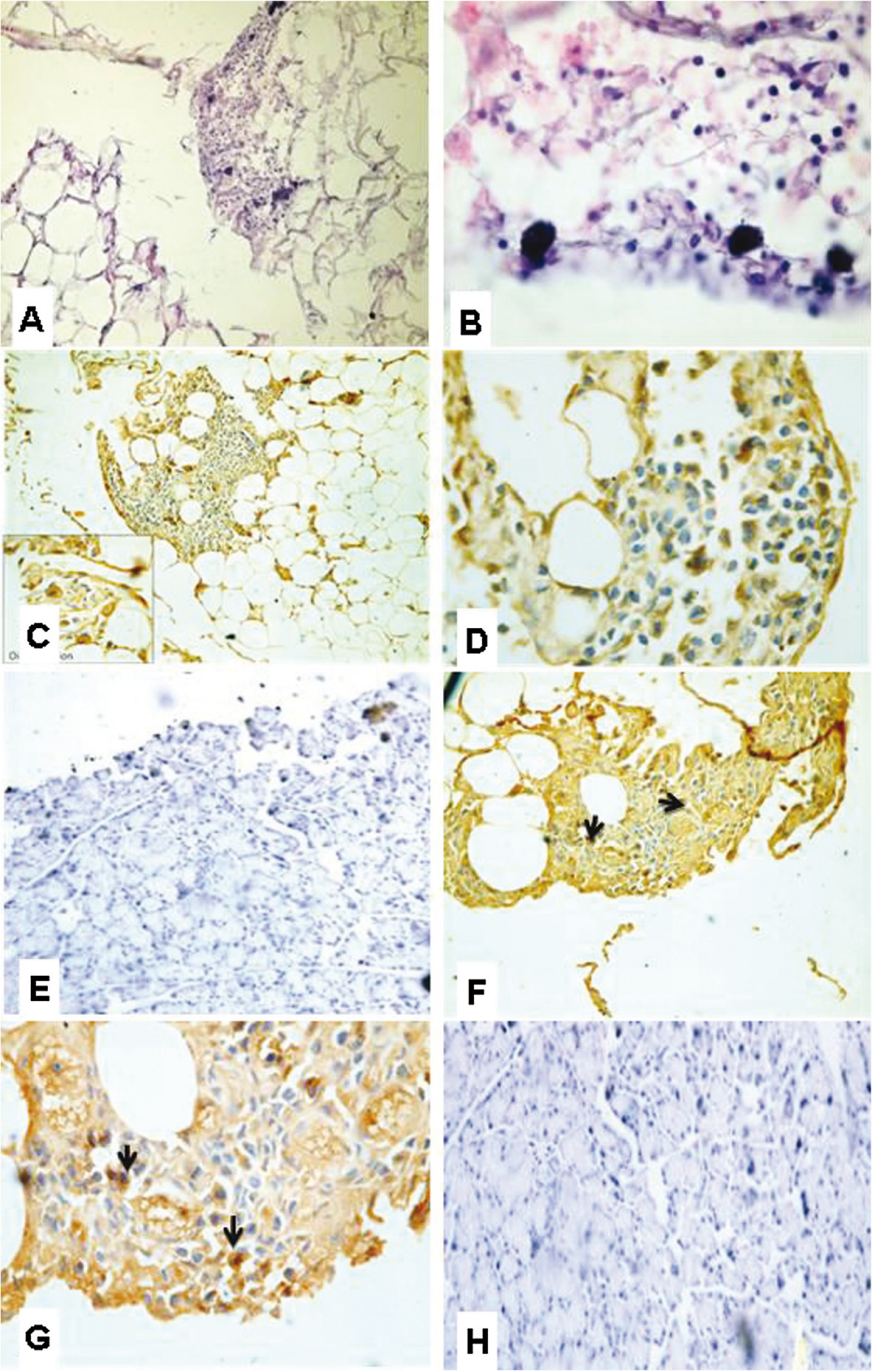
**Micrographs of diabetic rat pancreas (fed NCD) after 2 months from the onset of diabetes. (A)** Rat pancreas stained with H&E showed pancreatic tissue infiltrated by adipose tissue containing collections of primitive cells, but absence of pancreatic islets (X200) and **(B)** Few scattered larger cells with the appearance of lipoblasts scattered within these primitive cells (X400) **(C)** &**(D)** Diabetic rat pancreas stained with insulin antibody showed few large insulin positive cells in the adipose tissue with no evidence of islets in pancreatic tissue and no positivity for insulin (X200 and X1000,respectively) **(E)** Diabetic rat pancreas stained with insulin antibody showed no evidence of islets in pancreatic tissue and no insulin positivity (X200) **(F)** (X200) &**(G)** (X1000)Diabetic rat pancreas stained with CD 105 antibody showed positive CD105 cells in the adipose tissue. **(H)** Diabetic rat pancreas stained with CD 105 antibody showed no evidence of CD 105 positive cells in pancreatic tissue (X200).

Histological examination of the H&E-stained paraffin sections of the pancreas of diabetic rats fed with NCD after 2 months from the onset of diabetes showed pancreatic tissue infiltrated by adipose tissue with absence of islets of Langerhans and collections of oval-round small cells with minimal cytoplasm and dark nuclei in adipose tissue (primitive cells) (Figure 6A). The rat pancreas also showed few scattered larger cells with the appearance of lipoblasts having more abundant bubbly pale cytoplasm scattered within these primitive cells (Figure 6B). The rat pancreas stained with insulin antibody showed few large insulin positive cells in the cell collections in the adipose tissue (Figure 6C) and cells are larger than the rest with abundant cytoplasm with small round central dark nucleus (Figure 6D) but no evidence of islets in pancreatic tissue and no positivity for insulin (Figure 6E). The rat pancreas stained with CD 105 antibody showed scattered positive cells in the small blue cell collections in the adipose tissue (Figure 6F and G), but no evidence of CD 105 positive cells in pancreatic tissue (Figure 6H).

**Figure 7:**
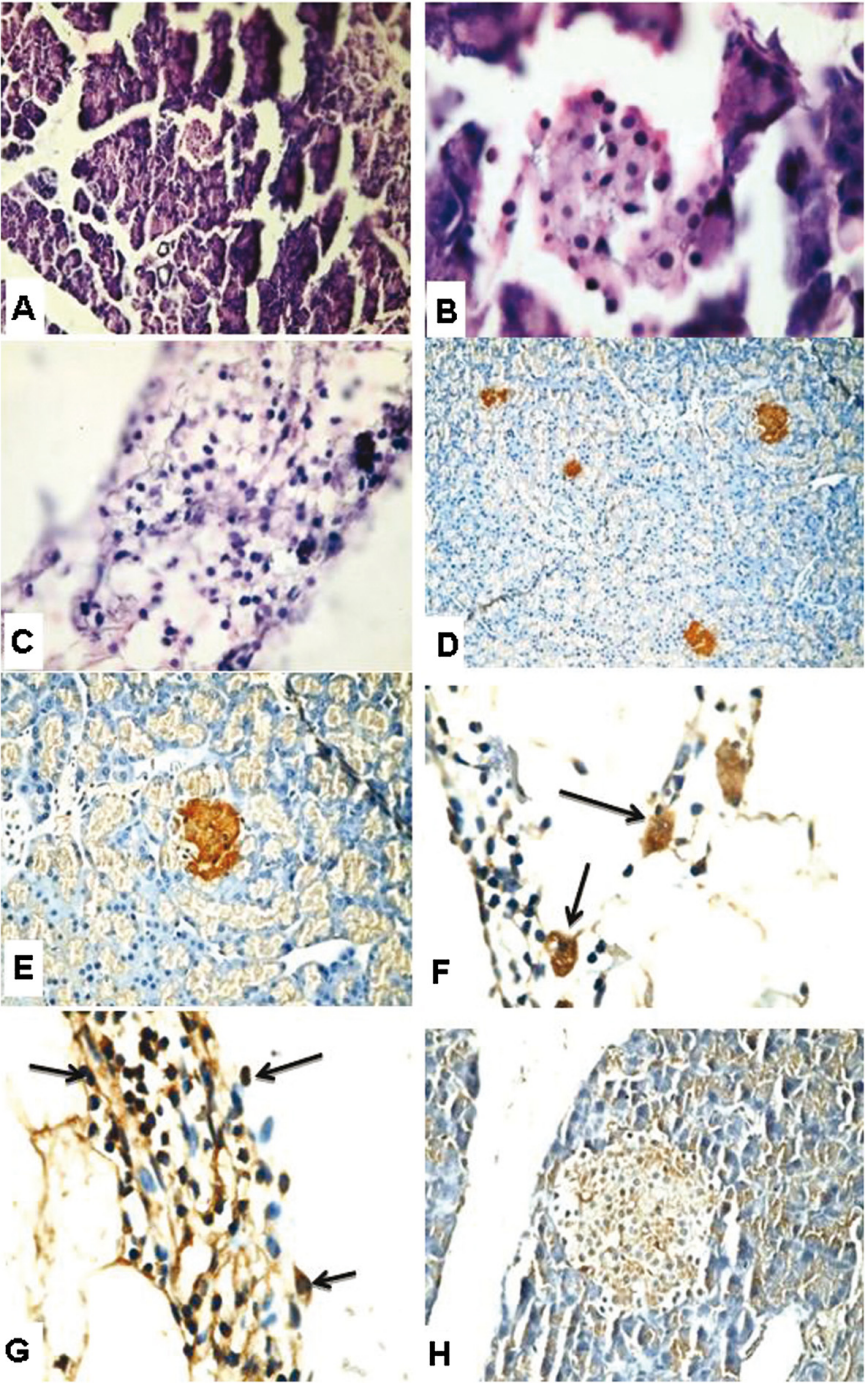
**Micrographs of diabetic rat pancreas (fed NCD) stained with after 3 months from the onset of diabetes. (A) **&**(B)** Rat pancreas stained with H&E showed more developed pancreatic tissue with evidence of small sized islets of Langerhan (X100 and X1000, respectively) **(C)** Collections of primitive cells in nearby adipose tissue and more abundant lipoblasts (X1000). **(D)** (X200) &**(E)** (X400) Diabetic rat pancreas stained with insulin antibody showed positive insulin producing cells in islets. **(F)** More large insulin positive cells in the cell collections in the adipose tissue (X1000) **(G)** Diabetic rat pancreas stained with CD 105 antibody showed more positive cells in the adipose tissue mostly adjacent to pancreatic tissue (X1000) and **(H)** no evidence of CD 105 positive cells in the pancreatic tissue (X400).

After 3 months from induction of diabetes the H&E-stained paraffin sections of diabetic rats (fed NCD) demonstrated more developed pancreatic tissue with evidence of small sized islets of Langerhan (Figure 7A and B). The pancreas also showed collections of oval – round small primitive cells with minimal cytoplasm and dark nuclei in nearby adipose tissue. More scattered larger cells with more abundant pale foamy cytoplasm & central dark round nuclei (lipoblastic cells) are found scattered within these primitive cells (Figure 7C). Diabetic rat pancreas stained with insulin antibody showed positive cells in islets (Figure 7D and E) with more large insulin positive cells in the cell collections in the adipose tissue (Figure 7F). Diabetic rat pancreas stained with CD 105 antibody showed more positive cells in the small blue cell collections in the adipose tissue mostly adjacent to pancreatic tissue (Figure 7G), but no evidence of CD 105 positive cells in the pancreatic tissue (Figure 7H).

**Figure 8:**
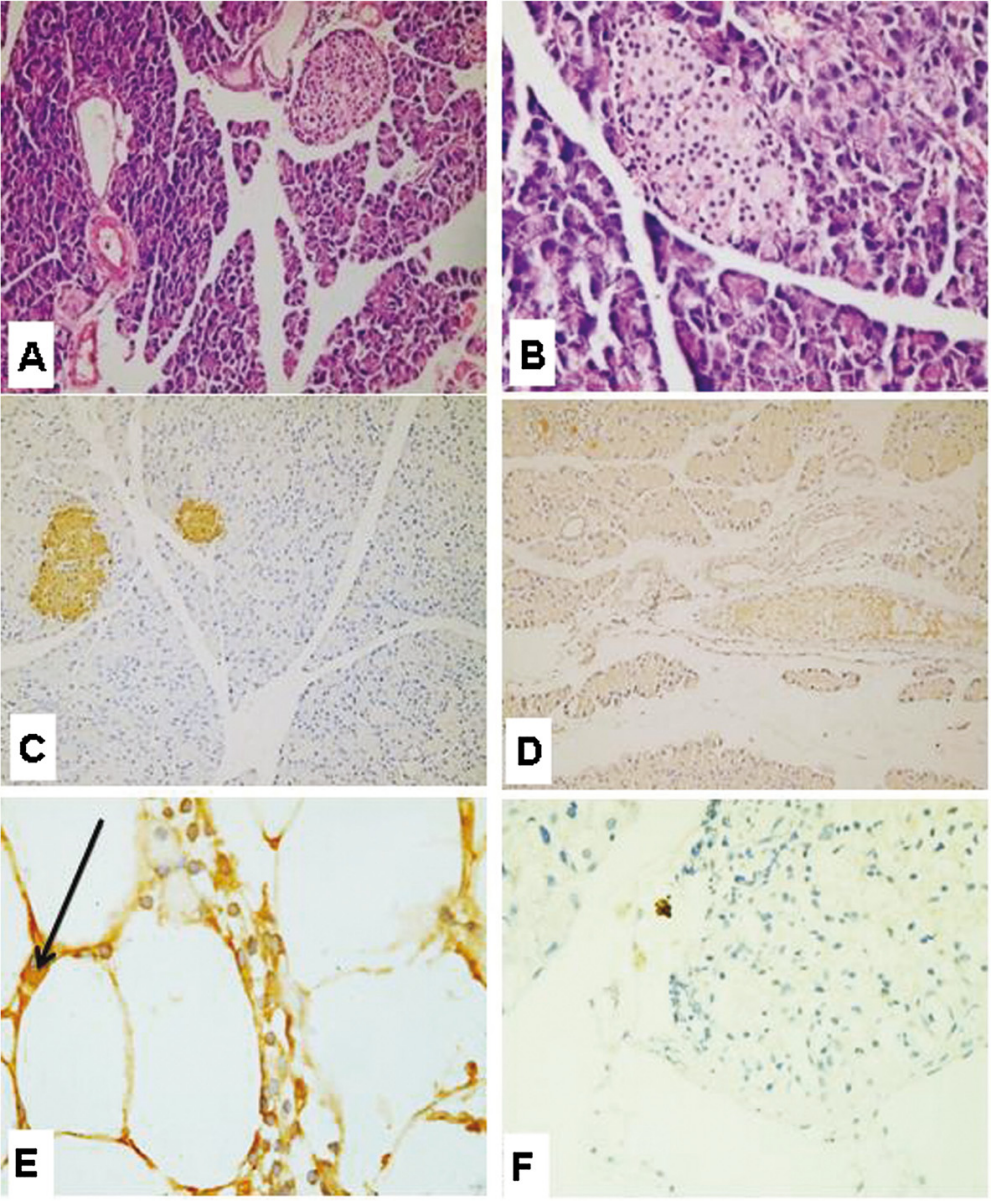
**Micrographs of diabetic rat pancreas (fed NCD) after 5 months from the onset of diabetes. (A)** (X200) &**(B)** (X400): Diabetic rat pancreas stained with H & E showed more developed pancreatic tissue with variable sized well developed larger sized islets situated in a paravascular pattern. **(C)** Diabetic rat pancreas stained with insulin antibody showed insulin positive islets of variable size (X200) **(D)** Diabetic rat pancreas stained with insulin antibody showed insulin negative adipose tissue (X200) **(E)** &**(F)** Diabetic rat pancreas stained with CD 105 antibody showed negative CD 105 in fat with an occasional rare positive cell (X1000 and X400,respectively).

After 5 months from induction of diabetes the H&E-stained paraffin sections of diabetic rats (fed NCD) showed more developed pancreatic tissue with variable sized well developed larger sized islets situated in a paravascular pattern with no evidence of primitive cell collections (Figure 8A and B). Diabetic rat pancreas stained with insulin antibody showed insulin positive islets of variable size (Figure 8C) and insulin negative adipose tissue (Figure 8D). Diabetic rat pancreas stained with CD 105 antibody showed negative CD 105 in fat with an occasional rare positive cell (Figure 8E and F).

**Figure 9:**
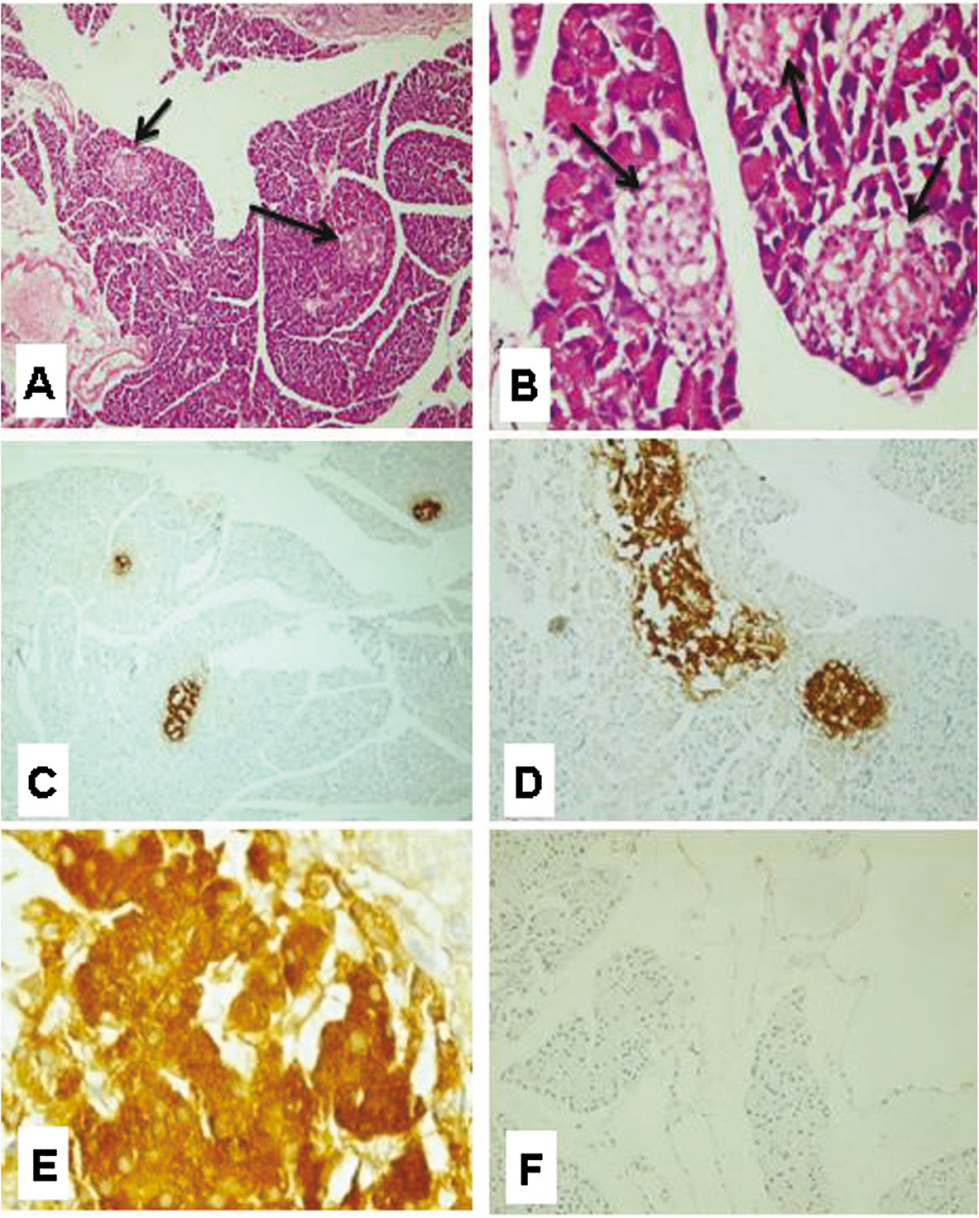
**Micrographs of diabetic rat pancreas (fed NCD) after 10 months from the onset of diabetes. (A)** (X100) &**(B)** (X400) Diabetic rat pancreas stained with H&E showed pancreatic tissue with increased amount of exocrine and endocrine components and many variable sized and shaped islets of Langerhans. **(C)** (X40), **(D)** (X200) &**(E)** (X1000) Diabetic rat pancreas stained with insulin antibody showed many variable sized & shaped islets with strong insulin positivity(brown) **(F)** Diabetic rat pancreas stained with CD 105 antibody showed no positive CD105 cells in pancreatic tissue or fat (X100).

Hematoxylin and Eosin sections of diabetic rat pancreas (fed NCD) after 10 months from induction of diabetes showed pancreatic tissue with increased amount of exocrine and endocrine components and many variable sized and shaped islets of Langerhans (Figure 9A and B). Diabetic rat pancreas stained with insulin antibody showed many variable sized & shaped islets with strong insulin positivity (Figure 9C, D and E). Diabetic rat pancreas stained with CD 105 antibody showed no positive CD105 cells in pancreatic tissue or fat (Figure 9F).

## Discussion

Effective management of diabetes is a pivotal global need that is yet to be established. Modern drugs, including insulin and other hypoglycemic agents, control the blood glucose level only when they are regularly administered, but these treatments are tedious and tend to pose several clinical challenges [24]. However, medicinal herbs may offer a similar degree of efficacy without so many troublesome side effects.

The effectiveness of curcumin in reducing secondary complications in STZ induced diabetic animals has been previously reported [25]. Moreover, curcumin has been demonstrated in prevention of isolated beta cell death and dysfunction induced by STZ [26,27]. Several studies also highlight its benefit as a hypoglycemic agent [16-20] however; a limited number of reports present the importance of curcumin in improvement of pancreatic islets in diabetes. In the present study, we would like to evaluate the antidiabetic effect of a novel curcumin derivative and its effect on pancreatic islet regeneration in type I diabetes-induced by STZ.

Diabetes in this study was induced by STZ, an alkylating agent that causes DNA damage in beta-cells and subsequent inhibition of insulin biosynthesis and secretion [28]. Our data showed that induction of diabetes by STZ led to a significant increase of plasma glucose and a significant decrease in plasma insulin and C-peptide levels compared to the basal levels. It has been reported that destruction of β-cells by streptozotocin remains as such incomplete that the resulting rats are hyperglycemic but do not die [29]. This view goes well with the currently observed minimal insulin secretion in untreated diabetic mice.

Our results demonstrated that the plasma glucose levels in diabetic rats decreased gradually while the insulin and C-peptide levels increased gradually although still, within the diabetic range until 4 months after induction of diabetes. This was followed by a more significant decrease in plasma glucose and a more significant increase in plasma insulin and C-peptide levels which continued for 10 months after induction of diabetes reaching almost normal basal levels.

These results could be explained by the fact that insulin-secreting pancreatic beta-cells proliferate in response to increasing demand for insulin and also after physiological injury [30-32]. It is generally accepted that beta-cells have a finite life span and that dying beta-cells are continuously replaced [33,34]. Indeed, there are clinical case-reports of beta-cell regeneration enabling the complete recovery from type 1 diabetes [35]. However, in the majority of patients, the reported level of recovery is not sufficient to cure, or even maintain glucose homeostasis [30,32].

Several reports of functional pancreatic beta-cell regeneration in murine models [36,37] has generated much excitement and controversy [38,39].

Curcumin is a naturally occurring yellow pigment powder extracted from the roots of the *Curcuma longa* plant (Turmeric). Curcumin has been shown to exhibit anti inflammatory [40], antioxidant [12], anti-tumor [41] and anti-diabetic activities [16,17]. Furthermore, curcumin has been demonstrated in protecting isolated islet against streptozotocin (STZ)-induced oxidative stress by scavenging free radicals [26]. However, its efficacy in ameliorative pancreatic islets from STZ-induced diabetic mice is still lacking.

Oral administration of NCD for forty days to diabetic rats significantly reduced the plasma glucose, significantly increased plasma insulin and C-peptide compared to the diabetic rats. The plasma insulin and C-peptide levels continued to increase for ten months from the onset of diabetes reaching levels significantly higher than the basal level.

The results of the current study are in accordance with the work of others who reported that administration of oral curcumin to diabetic mice or rats fed with curcumin resulted in a significant decrease in the blood glucose level when compared with the diabetic group [19,42-44].

Histopathological examination of diabetic rat pancreas 2 months after induction of diabetes, revealed mostly exocrine pancreatic tissue with no islets of Langerhans, minimal adipose tissue infiltration and localized lymphocytic infiltrates. Also, pancreatic tissues and adipose tissues showed no evidence of insulin or CD105 positive cells. Diabetes type I is an autoimmune disease that is characterized by selective destruction of insulin-producing β-cells found in the pancreatic islets of Langerhans [45]. The development of diabetes is initiated with insulitis, in which leukocytes migrate to and invade the islets. This is followed by an overt, insulin deficient diabetes phase that is distinguished by destruction of the majority of β-cell [2]. However, after 6 months of induction of diabetes, rat pancreas showed the appearance of small well formed islets and positive insulin cells but no CD105 positive cells. This resulted in the observed gradual increase in plasma insulin and decrease in plasma glucose levels, suggesting that the expansion of beta cell mass compensated for increased insulin demands in order to control blood glucose homeostasis [46].

Each tissue or organ is believed to contain a small sub-population of cells that is capable of self-renewal and has the ability to give rise to each mature cell type [47]. Thus, one of the most promising sources of beta cells might be pancreatic stem cells. While several studies have shown the existence of pancreatic stem cells [48-50], these cells have not yet been isolated from the pancreas in a pure form.

Early studies by Slack [51] indicated the failure of islet regeneration following treatment with streptozotocin or alloxan and suggested that these drugs target, in addition to the differentiated β-cells, the potential stem or transit cells. Contrary to this report, more recent studies have shown that streptozotocin does not destroy the intra-islet stem cell reserve in experimentally-diabetic animals [52]. In support, Ianus et al. [53] demonstrated that bone marrow cells can differentiate into functionally competent pancreatic β-cells; thereby rationalizing for the cell-based approach in treatment of diabetes.

Histopatological examination of diabetic rat pancreata (fed NCD for 40 days) was performed after 2, 3, 5 and 10 months from the onset of diabetes.

Two months from the onset of diabetes, histopathological examination of rat pancreas showed the appearance of collections of primitive cells in adipose tissue infiltrating the pancreas. Also, large insulin positive cells were found in the adipose tissue with absence of islets in pancreatic tissue and no positivity for insulin. To identify these cells, rat pancreas was stained with CD 105 antibody which showed scattered positive cells in the small blue cell collections in the adipose tissue while no evidence of CD 105 positive cells in pancreatic tissues was observed.

Crisan et al. [54] have found that multipotent mesenchymal stem cells exist in many different human organs. To support this fact, it has been shown that MSCs are not confined to bone marrow and can also be found in placenta, dental pulp, tendons, skeletal muscle, fat, umbilical cord blood and amniotic fluid [55-59].

After 3 months from the onset of diabetes, rat pancreas showed more developed pancreatic tissue with evidence of small sized islets of Langerhan. Collections of small primitive cells in nearby adipose tissue were also seen. Rat pancreas stained with insulin antibody showed positive cells in islets. More large insulin positive cells were found in the cell collections in the adipose tissue. Staining with CD 105 antibody showed more positive cells in the adipose tissue mostly adjacent to pancreatic tissue. However, rat pancreas stained with CD 105 antibody showed no evidence of CD 105 positive cells in the pancreatic tissue.

After 5 months from the onset of diabetes, rat pancreas showed more developed pancreatic tissue with variable sized well developed larger sized islets situated in a paravascular pattern with disappearance of primitive cell collections. Rat pancreas stained with insulin antibody showed insulin positive islets of variable size with disappearance of insulin positive cells in adipose tissue. Rat pancreas stained with CD 105 antibody showed negative CD 105 in fat with an occasional rare positive cell.

After 10 months from the onset of diabetes, rat pancreas showed pancreatic tissue with increased amount of exocrine and endocrine components and many variable sized and shaped islets of Langerhans. Staining with insulin antibody showed many variable sized & shaped islets with strong insulin positivity and no CD 105 positive cells in pancreatic tissue or fat.

Histopatholgical and immunohistochemical examination reported the appearance of stem cells in the adipose tissue infiltrating the pancreatic tissues in diabetic rat receiving the NCD. These cells were positive for CD105. This coincides with the work of Moniri et al. [60] who reported that pancreas-derived MSCs exhibited positive expression of CD44, CD73, CD95, and CD105 and Xiao-Jie et al. [61] who reported that CD105 can be used as a relatively specific marker for the selection of adipose-derived stem cells.

It is known that regeneration processes are needed to maintain the critical cell mass which is necessary to maintain organ homeostasis. The potential of replication is limited in the case of pancreatic β-cells. However, in a population of normal adult pancreatic islet cells, the number of β-cells actually undergoing cell division is small, measured to be between 0.5 and 2% [62]. Only in recent years has it been revealed that the pancreas can indeed undergo repair following damage, a process known today as regeneration.

A study by Yamamoto et al. [63] showed that the fetal pancreas treated with STZ has the ability to regenerate β-cells *in vivo*. Once it was established that pancreas can regenerate, researchers started to look at the factors and stimuli that instruct pancreatic tissue to undergo regeneration.

The pancreas has been reported as the organ with the lowest levels of antioxidant enzymes; consequently, pancreatic β-cells are exceptionally vulnerable to detrimental actions of oxidative stress [64]. The cytotoxic, diabetogenic action of streptozotocin is mediated mostly by reactive oxygen species [65]. Accordingly, curcumin, which possess antioxidant [12] and anti inflammatory properties [13] could play a role in reversing the effects of experimental streptozotocin-induced diabetes.

Curcumin abates oxidative stress due to reactive oxygen species and lipid-peroxidation by reducing hyperglycemia, and enhancing endogenous antioxidant machinery (glutathione peroxidase, catalase, superoxide-dismutase, and reduced glutathione, and GSH). Abdel Aziz et al. [18,19,44] studied the antidiabetic and antioxidant effects of curcumin and NCD on isolated pancreatic islets and in type -1 diabetes. These studies reported that NCD increased HO-1 gene expression and HO activity in the pancreas. NCD also improved the oxidative status, protected and enhanced endogenous defenses directly proved by decreasing lipid peroxides (malondialdehyde) in pancreas & liver.

This certainly protects residual pancreatic islets and newly bone marrow transplantation-generated ones against cellular damage evoked by reactive oxygen species or advanced-glycation end products [66,67].

In addition, curcumin obliterates inflammation and immune response; as evident by its ability to suppress production of the cytokines, tumor necrosis factor (TNF)-α and interleukin (IL)-1β [68]. This certainly creates a favorable systemic and pancreatic environment to foster bone marrow transplantation and islet neogenesis. Accordingly, administration of curcumin; as an established anti-inflammatory and immune modulatory drug; would likely boost and preserve the process of islet regeneration; which was evidently proven true in this study.

Previous studies have indicated that cucumin plays an important role in regulation of cell differentiation. Thaloor et al., (1999) [69] demonstrated that curcumin is involved in stimulation of muscle regeneration after traumatic injury by directly inducing proliferation and differentiation of muscle precursor cells Other studies have also shown that curcumin induces neurogenesis, synaptogenesis and migration of neural progenitor cells *in vitro* and *in vivo*[70,71]. Recently, Mujoo et al. (2012) [72] reported that curcumin induces differentiation of embryonic stem cells through possible modulation of nitric oxide –cyclic GMP pathway.

## Conclusion

Our current data suggest that the NCD can significantly ameliorate experimental type 1diabetes. Our study provides clear evidence of pancreatic islets regeneration in response to treatment of diabetic rats with the NCD for forty days. This could be attributed to the anti –inflammatory and antioxidant effects of curcumin and thus creates a favorable systemic and pancreatic environment to foster islet neogenesis. Also, the role of curcumin in cell proliferation and differentiation of stem cells may be involved.

## Competing interests

The authors declare that they have no competing interests.

## Authors' contributions

MT contributes in study design, manuscript drafting and critical discussion. MF contributes in study design, and critical discussion. AR contributes in preparation of the novel curcumin derivative. SM contributes in histopathological and immunohistochemical work and analysis. MA contributes in analysis and manuscript drafting. HHA contributes in study design, practical work, manuscript drafting and critical discussion. HHF contributes in analysis and manuscript drafting. FM contributes in practical work, manuscript drafting and critical discussion. All authors read and approved the final manuscript.
